# Psychometric properties of the Dresden Body Image Questionnaire: A multiple-group confirmatory factor analysis across sex and age in a Dutch non-clinical sample

**DOI:** 10.1371/journal.pone.0181908

**Published:** 2017-07-26

**Authors:** Mia Scheffers, Marijtje A. J. van Duijn, Ruud J. Bosscher, Durk Wiersma, Robert A. Schoevers, Jooske T. van Busschbach

**Affiliations:** 1 Windesheim University of Applied Sciences, School of Human Movement and Education, Zwolle, The Netherlands; 2 University of Groningen, Department of Sociology, Groningen, The Netherlands; 3 University of Groningen, University Medical Center, Rob Giel Research Centre, Groningen, The Netherlands; 4 University of Groningen, University Medical Center, Department of Psychiatry, Research School of Behavioural and Cognitive Neurosciences (BCN), Interdisciplinary Center for Psychopathology and Emotion regulation (ICPE), Groningen, The Netherlands; Georgetown University Medical Center, UNITED STATES

## Abstract

**Background:**

Body image has implications for psychosocial functioning and quality of life and its disturbance is reported in a broad range of psychiatric disorders. In view of the lack of instruments in Dutch measuring body image as a broad concept, we set out to make an instrument available that reflects the multidimensional character of this construct by including more dimensions than physical appearance. The Dresden Körperbildfragebogen (DBIQ, Dresden Body Image Questionnaire) particularly served this purpose. The DBIQ consists of 35 items and five subscales: body acceptance, sexual fulfillment, physical contact, vitality, and self-aggrandizement. The main objective of the present study was to evaluate the psychometric properties of the Dutch translation of the Dresden Body Image Questionnaire (DBIQ-NL) in a non-clinical sample.

**Methods:**

The psychometric properties of the DBIQ-NL were examined in a non-clinical sample of 988 respondents aged between 18 and 65. We investigated the subscales' internal consistency and test-retest reliability. In order to establish construct validity we evaluated the association with a related construct, body cathexis, and with indices of self-esteem and psychological wellbeing. The factor structure of the DBIQ-NL was examined via confirmatory factor analysis (CFA). The equivalence of the measurement model across sex and age was evaluated by multiplegroup confirmatory factor analyses.

**Results:**

Confirmatory factor analyses showed a structure in accordance with the original scale, where model fit was improved significantly by moving one item to another subscale. Multiple group confirmatory factor analysis across sex and age demonstrated partial strong invariance. Internal consistency was good with little overlap between the subscales. Temporal reliability and construct validity were satisfactory.

**Conclusion:**

Results indicate that the DBIQ-NL is a reliable and valid instrument for non-clinical subjects. This provides a sound basis for further investigation of the DBIQ-NL in a clinical sample.

## Introduction

The term ‘body image’ has been used to describe a variety of body-related phenomena, including perceptions, cognitions, affects, and awareness with regard to the body [1,2]. Unlike the term seems to suggest, not only the way we evaluate our appearance is part of our body image, but also our attitude towards bodily experiences in interaction with others, sense of body ownership [3] and our evaluation of our body in terms of functionality and vitality [4]. Aspects of body image have impact on psychosocial functioning [5, 6] and its disturbances are associated with poorer psychological adjustment in non-clinical samples [7–10]. Negative or disturbed body image has also been reported in a broad range of psychiatric disorders [11, 12]. Disturbed body image in eating disordered female patients has been extensively researched and documented [13–15]. There is emerging evidence that mood disorders [16], anxiety disorders [17], trauma-related disorders [18–20], sexual disorders [21, 22] and schizophrenia [23] are also associated with negative or disturbed body image. The overall impression is that body image is affected in a diverse range of mental health problems. However, despite the increasing awareness and recognition in clinical practice of body image problems in other than appearance-related psychopathologies, sound measurement of body image providing evidence of this phenomenon is still scarce in most psychiatric disorders.

To date, the Body Attitude Test is the sole well-researched self-report instrument measuring body image available in Dutch [24, 25]. However, this instrument is specifically developed for measuring body attitude in anorexic women. In view of the lack of multi-purpose instruments in Dutch, we set out to make an instrument available that reflects the multidimensional character of this construct by including more dimensions than physical appearance. As our aim was to gain insight in the way psychopathology and body image are associated, this instrument needed to be suitable for both a broad-ranged clinical population as well as for a non-clinical population so as to facilitate comparisons. The Dresdner Körperbildfragebogen (DBIQ, Dresden Body Image Questionnaire) [26, 27] particularly serves this purpose. The DBIQ, consisting of 35 items, does not cover all aspects that form part of the umbrella term body image, but focuses on thoughts, beliefs, and conceptual aspects of patient’s body experiences in five different domains: body acceptance, sexual fulfillment, the evaluation of physical contact, experienced vitality, and self-aggrandizement, a measure of how the body is actively used in social interactions to enhance self-esteem. The importance of the dimensions physical contact and sexuality is largely unknown, although they are often mentioned by patients as problematic [28, 29]. Furthermore, in a factor analytic evaluation of a preliminary version of the DBIQ, body contact as well as sexuality emerged as separate factors [26]. Clinical relevance together with the psychometric indication of the importance of these aspects, make its inclusion worthwhile. The first evaluation of the psychometric qualities of the original DBIQ in a clinical sample was promising [26].

The main objective of the present study was to evaluate the psychometric properties of the Dutch translation of the DBIQ (DBIQ-NL) in a non-clinical sample. First, we investigated the subscales’ internal consistency and test-retest reliability. In order to establish construct validity of the DBIQ-NL we evaluated the association with specific related constructs, namely body satisfaction, comfort with touch and fatigue, expecting high correlations for the DBIQ-NL with these measures. Furthermore, we evaluated associations with general indices of psychosocial wellbeing and self-esteem, expecting moderate to high correlations with these measures. The factor structure of the DBIQ-NL was examined via confirmatory factor analysis (CFA).Exploration of differences regarding gender and age formed the second objective of our study, since both factors are known to affect body image. Gender differences in body image have received considerable attention [30–32]. Research shows that women are generally more preoccupied and dissatisfied with their bodies than men [33]. Gender differences with respect to specific issues such as physical contact and sexual fulfillment, represented by separate subscales in the DBIQ, also were deemed worth investigating. Based on results from a Flemish version of the DBIQ in a students’ sample [34], it might be hypothesized that no gender differences exist with regard to physical contact. With regard to gender differences in reported sexual fulfillment it might be hypothesized, based on results from Dutch population surveys [35], that sexual fulfillment is higher in men than in women. Contrary to gender differences, age effects on body image are still poorly researched [31, 36]. Since younger aged samples are overrepresented in body image research, comparisons of different age groups are scarce. Krauss et al. [37] have noted a clear need for research on body image in middle-aged and older adults. The non-clinical sample enables further investigation of specific aspects of body image in middle-aged adults and comparison with a younger group.

Group comparisons of scale scores are only meaningful in case of measurement invariance across groups. Therefore, the equivalence of the measurement model across sex and age using Multiple-Group Confirmatory Factor Analyses (MG-CFA) was evaluated in this study as well, extending the work by Pöhlmann et al. [26, 27].

## Method

### Participants

Data were obtained from two samples with a total of 988 (sample 1, *n* = 761; sample 2, *n* = 227) respondents between 18 and 65 years old, consisting of 583 (433; 150) women and 403 (326; 77) men (sex was unknown for two respondents). In both samples age showed a bimodal distribution and was therefore divided in two categories, younger than 38 years, and 38 years and older, based on visual inspection (sample 1: < 38, *n* = 540; ≥ 38: *n* = 221; sample 2: < 38, *n* = 151; ≥ 38: *n* = 76; see S1 Fig. for more information on distribution of age in the two samples). Women and men were equally distributed across the two categories. *M*_age_ of sample 1 was 30.90 (*SD*_age_ = 13.62), of sample 2 33.28 (*SD*_age_ = 13.22). The distinction between the two samples was made based on the moment of recruitment (see procedure). Sample 2 was offered a partly different set of extra instruments compared to sample 1.

### Measures

#### Dresden Body Image Questionnaire

The Dresden Body Image Questionnaire (DBIQ) [26, 27] is a 35-item scale (see Table 1) with positively and negatively worded items (reversely coded) that consists of five subscales: body acceptance (e.g., “I wish I had a different body”), vitality (e.g., “I am physically fit”), physical contact (e.g., “I do not like people touching me”), sexual fulfillment (e.g., “I am very satisfied with my sexual experiences“), and self-aggrandizement (e.g., “I use my body to attract attention”*)*. Level of agreement is scored on a 5-point Likert scale ranging from 1 = *not at all* to 5 = *fully*. Higher scores indicate a more positive body image.

**Table 1:** Dresden Body Image Questionnaire (DBIQ), English version^a^.

1.a	I move gracefully.
2.v	I often feel physically run down. (R)
3.v	I lack energy and motivation. (R)
4.s	I experience intense and pleasurable feelings during sex.
5.p	Physical contact is important for me to express closeness.
6.v	I often feel physically exhausted. (R)
7.a	There are lots of situations in which I feel happy about my body.
8.v	I am physically fit.
9.s	I am very satisfied with my sexual experiences.
10.a	Other people find me attractive.
11.p	I look for physical intimacy and affection
12.a	I like my body.
13.a	I find it pleasant and exhilarating when someone looks at me attentively.
14.v	I have lots of energy.
15.a	I choose clothing that hides the shape of my body. (R)
16.s	I think sex is an important part of life.
17.v	I am in good physical condition.
18.a	I often feel uncomfortable about my body. (R)
19.p	I do not like people touching me. (R)
20.a	I feel more valued when someone pays attention to my body.
21.s	I am able to lay aside my inhibitions in sexual situations.
22.p	I like it when people put their arms around me.
23.a	I wish I had a different body. (R)
24.p	I consciously avoid touching other people. (R)
25.a	I am satisfied with my appearance.
26.v	I quickly reach my physical limits. (R)
27.s	I am able to enjoy my sexuality.
28.a	If I could change something about my body, I would do it. (R)
29.a	My body is expressive.
30.p	I only allow a few people to touch me. (R)
31.a	I use my body to attract attention.
32.v	I am physically strong and resilient.
33.a	I like showing my body.
34.a	I like to be the centre of attention.
35.s	My sexual experiences are satisfying.

Note: R = scored in the reversed direction. a = subscale self-aggrandizement; b = subscale body acceptance; p = subscale physical contact; s = subscale sexual fulfillment; v = subscale vitality.

a. Dresdner Körperbildfragebogen (DKB-35), original version in German; for Dutch version, see S1 Table.

The development of the DBIQ was based on factor analytic evaluation of three German questionnaires measuring body image [38], namely the “Fragebogen zum Körperbild” (FKB-20) [39], the “Fragebogen zur Bewertung des eigenen Körpers” (FBeK) [40, 41] and the “Frankfurter Körper Konzept Skalen” (FKKS) [42].

In a German non-clinical sample [27] (*n* = 418), Cronbach’s *α* for the subscales were: body acceptance .93, vitality .94, physical contact .83, sexual fulfillment .91, and self-aggrandizement .81. Correlations between the subscales varied between *r* = .37 (sexual fulfillment and self-aggrandizement) and *r* = .65 (body acceptance and vitality), indicating the overlap between the subscales to be small to medium. A confirmatory factor analysis was conducted [26] in a sample of 560 German patients with psychosomatic disorders (*CFI* = .90; *RMSEA* = .06, other fit indices not available). A study on 505 students (*M* = 21.64, *SD* = 2.14) using a Flemish Dutch translation [34], somewhat different from the translation presently used, reported Cronbach’s α for the subscales between .77 and .90. Correlations between the subscales varied between *r* = .13 and *r* = .59. In the present Dutch sample Cronbach’s *α* for the subscales varied from *α* = .83 for self-aggrandizement to *α* = .92 for sexual fulfillment. Correlations between the subscales varied between *r* = .31 (vitality and physical contact) to *r* = .65 (physical contact and sexual fulfillment).

The Dutch translation of the DBIQ (DBIQ-NL, see S1 Table) was performed by using the parallel blind technique [43]. First, three bilingual translators separately performed a translation. The translations were then compared and differences were discussed until agreement was reached.

#### Body Cathexis Scale

The Body Cathexis Scale (BCS), here used to establish the association between the total score of the DBIQ with body satisfaction, was originally developed by Secord and Jourard [44] to assess the degree of satisfaction with parts and processes of the body. The original scale has 46 items, but most recent studies utilize a 40-item version [45]. Subjects evaluate body characteristics according to a 5-point Likert scale, ranging from *strongly negative* to *strongly positive*, with higher scores reflecting greater body satisfaction. Although some authors [46] objected to the use of anatomical as well as physiological aspects in the BCS, we follow Orlandi et al. [47], who state that the BCS is a useful instrument to address satisfaction with the body and judge the emphasis on bodily functions next to body parts to be an advantage. This is in line with recent studies [48, 49] stressing the importance of describing the body in functional terms. The validity and reliability of the Dutch version [50] are satisfactory. Cronbach’s *α* for the present sample was .95.

#### Comfort in touch (subscale of the Body Investment Scale)

Since no reference is made in the BCS to physical contact, a subscale of the Body Investment Scale (BIS) [51] was used to establish the association with the subscale ‘physical contact’ of the DBIQ. The BIS was developed to assess emotional investment in the body and consists of 24 items scored on a 5-point Likert scale ranging from *strongly disagree* to *strongly agree* with higher scores for more emotional investment. The subscale physical contact of the BIS comprises of six items with statements like “I enjoy physical contact with other people”. The BIS has adequate psychometric characteristics [51, 52]. In the present study a Cronbach’s α of .78 was found for the subscale ‘comfort in touch’.

#### Checklist Individual Strength

As the BCS does not include items on vitality the Checklist Individual Strength (CIS) [53] was used to establish the construct validity of this subscale of the DBIQ. The CIS is an originally Dutch language 20-item self-report questionnaire capturing fatigue in four dimensions: subjective experience of fatigue (“I feel tired”), reduction in motivation (“I feel no desire to do anything”), reduction in activity (“I don’t do much during the day”) and reduction in concentration (“My thoughts easily wander”) and has been used in a broad range of groups: healthy subjects, diverse groups of working adults, people with chronic fatigue as well as people with multiple sclerosis [54]. By adding the four dimensions a CIS total score can be calculated. Respondents rate the extent to which each statement is true for them on a 7-point Likert scale ranging from *Yes*, *that is true* to *No*,*that is not true*. A higher score indicates more fatigue. Fatigue as measured with the CIS may be regarded as the opposite of vitality. In their description and evaluation of measures of fatigue, Hewlett et al. [55] evaluated the CIS as a useful generic scale and research tool, with no significant respondent or administrative burden. The CIS has demonstrated satisfactory psychometric properties [54, 56]. For the present study, Cronbach’s α was .94.

#### Rosenberg Self-esteem Scale

The Rosenberg Self-Esteem Scale (RSES) [57], Dutch version [58], is a brief 10-item measure of global self-esteem that evaluates one’s overall feelings of self-worth using a 4-point scale (1 = *strongly disagree*, 4 = *strongly agree*). Scores range from 10 to 40, with higher scores reflecting higher self-esteem. The validity and reliability of the Dutch version are satisfactory [59]. The internal consistency in the present study was .87.

#### Outcome Questionnaire

The Outcome Questionnaire (OQ-45) [60] is a 45-item scale measuring three domains of psychological well-being: subjective discomfort (“I feel no interest in things”), interpersonal relations (“I am satisfied with my relationships with others”), and social role performance (“I feel that I am doing well at work/school”). The OQ-45 is rated on a 5-point Likert scale ranging from *never* to *almost always* with higher scores for more distress. In a non-clinical group, Cronbach’s *α* was .93 for the original total scale [60] and .92 in the Dutch version [61]. Cronbach’s *α* in the present study was .90.

### Procedure

The research was conducted in agreement with the VU University Amsterdam guideline for research for educational purposes, allowing students to collect data with the use of questionnaires in healthy groups of respondents when participation is voluntary and data are analysed anonymously. We consulted the Medical Ethics Review Committee of VU University about the study, and the committee waived the requirement for ethical approval.

Data collection was done during an undergraduate course in measurement and statistics at the Faculty of Human Movement Sciences, VU University Amsterdam, resulting in a convenience sample. Students in two successive courses were encouraged to forward an e-mail with a link to the questionnaires to individuals in their personal network. No participatory incentives were offered. Participants completed all questionnaires without personal details through a secured online system and with all materials removed from this system after completing the data collection; data analysis was done anonymously. Information about the aim of the study and the voluntary and anonymous nature of participation was given before participants entered the study. In this way consent was secured when participants completed the questionnaire and no formal informed consent was necessary.

Of the respondents all were given the DBIQ-NL to fill out. Of the respondents included via students in the first course (sample 1 in Table 2) 361 (sample 1a) were asked to also complete the Body Cathexis Scale (BCS) [44], and 356 others (sample 1b) to complete the Rosenberg Self-Esteem Scale (RSES) [57], the Checklist Individual Strength (CIS) [53] and the subscale ‘comfort in touch’ of the Body Investment Scale (BIS) [51]. Respondents (*n* = 227) recruited by the students in the second course (sample 2) also completed the Outcome Questionnaire (OQ-45) [60] and the Rosenberg Self-esteem Scale. To assess temporal reliability, 56 respondents of sample 2 completed the DBIQ-NL a second time after 14 days. 44 respondents only completed the DBIQ-NL and did not fill in any additional questionnaires.

**Table 2:** Mean scores and mean differences sex and age DBIQ-NL samples and validation measures.

		full sample			women			men				age < 38			age ≥ 38			
Sample	(sub)scale	*n*	Mean	*SD*	*n*	Mean	*SD*	*n*	Mean	*SD*	*d*	*n*	Mean	*SD*	*n*	Mean	*SD*	*d*
1	DBIQ-NL	755^*^	3.66	0.45	430	3.56	0.44	323	3.79	0.43	0.53	537	3.71	0.44	218	3.53	0.45	0.40
1a	BCS	361	3.68	0.54	216	3.61	0.53	145	3.79	0.54	0.34	228	3.70	0.53	133	3.64	0.56	0.11
1b	RSES	356	3.26	0.47	191	3.15	0.45	163	3.39	0.44	0.54	272	3.25	0.47	84	3.28	0.44	0.07
1b	BIS-touch	356	3.77	0.61	191	3.73	0.56	163	3.81	0.66	0.13	272	3.80	0.63	84	3.66	0.54	0.24
1b	CIS	356	2.97	1.02	191	3.11	1.02	163	2.81	0.99	0.30	272	2.98	1.00	84	2.96	1.09	0.02
2	DBIQ-NL	227	3.57	0.39	150	3.53	0.39	77	3.64	0.40	0.28	151	3.59	0.40	76	3.53	0.38	0.15
	OQ-45	227	1.96	0.38	150	1.98	0.31	77	1.93	0.45	0.13	151	1.96	0.40	76	1.94	0.36	0.05
	RSES	225	3.32	0.46	149	3.26	0.45	76	3.44	0.45	0.40	149	3.31	0.48	76	3.34	0.41	0.07

Note: *d* = Cohen’s *d*; DBIQ-NL = Dresden Body Image Questionnaire, Dutch translation; OQ-45 = Outcome Questionnaire; RSES = Rosenberg Self-esteem Scale

BCS = Body Cathexis Scale; BIS-touch = subscale comfort with physical touch Body Investment Scale; CIS = Checklist Individual Strength.

* For six participants, no total scores were available.

### Data analysis

SPSS 17.00 for Windows was used for general statistical analyses. Mean differences between subgroups are expressed in Cohen’s *d* and considered large if > .80, moderate between .50 and .79 and small between .20 and .49 [62]. In calculating means we limited missing values to one for all subscales and two for total mean scores. Test-retest reliability was established by intraclass correlation (*ICC*). ICC > .75 was considered as excellent and between .40 and .75 as acceptable [63]. Construct validity was investigated by correlations. We expected a moderate correlation of body image total score with body satisfaction, because the DBIQ is aimed to measure a broader concept of body image. We also expected moderate correlations of body image total score with self-esteem and psychological well-being. Furthermore, high correlations are expected between the subscale ‘vitality’ of the DBIQ-NL and fatigue as measured with the CIS and between the ‘physical contact’ subscale of the DBIQ-NL and the comfort in touch subscale of the BIS.

The factorial structure of the translated version was tested by confirmatory factor analysis (CFA). Analyses were conducted with Mplus Version 5.1 [64], using the robust full-information maximum likelihood (MLR) estimator to correct for the skew distribution of several items and missing item responses [65]. In view of the sufficiently large sample size and focus on model selection and fit, the 5-point Likert items were treated as continuous measures [66]. Complete descriptives for all items used for the CFA are provided in S2 Table.

Because a five factor model was shown to be adequate for the German questionnaire, we investigated the fit of this model to the Dutch samples, aiming to obtain fit measures close to those of the German model. We could not reasonably expect equivalent fit measures as the study is not an exact replication study. The three essential changes with respect to Pöhlmann et al.’s [26] study, (1) translation of the items, (2) non-clinical samples, and (3) using a CFA model without correlated errors, were expected to lead to a decrease in model fit [67, 68].

Because each type of index provides different information about model fit [69], we chose to report a broad range of indices and also included standardized root mean square residual (SRMR) and Tucker Lewis index (TLI), in addition to the CFI and RMSEA reported in the CFA on the German items [26]. The RMSEA (Root Mean Square Error of Approximation) represents the fit of the estimated covariance matrix to the populations covariance matrix [70]. It is regarded as one of the most informative fit indices due to its sensitivity to the number of estimated parameters in the model and therefore favouring parsimonious models. As a rule of thumb, RMSEA values less than .08 suggest adequate model fit and RMSEA values less than .05 suggest good model fit [71]. The SRMR (Standardised Root Mean square Residual) is the standardized square root of the difference between the residuals of the sample covariance matrix and the hypothesised covariance model. An SRMR between .05 and .10 indicates an acceptable fit and values less than .05 indicate good fit [72]. The CFI (Comparitive Fit Index) [73] compares the sample covariance matrix with a null model of uncorrelated latent variables. The CFI is one of the most commonly reported fit indices due to being one of the measures least effected by sample size and is often reported together with the TLI (Tucker Lewis Index), a comparative fit index slightly differing from the CFI in its approach to sample size and handling of the effect of model complexity [69]. CFI and TLI values in the range between .90 and .95 may be regarded as indicative of acceptable model fit [69]. Although there is discussion on which fit-indices are the most relevant, it is now common practice to test the fit of the CFA with at least the ones used here. Thus, conclusions about the fit of the model can be based on the consistency between fit-indices. When fit-indices fall in marginal ranges, it is especially important to consider the consistency of the model fit as expressed by the various types of fit indices in tandem with the particular aspects of the analytic situation [69]. Inadequate fit measures are an indication of model misspecification. Modification indices can be used to adjust the model specification in order to improve model fit.

First, the five-factor model (without correlated errors) was estimated, using sample 1. Modification indices were inspected to detect possible improvements with respect to dimensionality. We refrained from including correlated errors in view of the multiple group confirmatory factor analysis (MG-CFA), which does not allow correlated errors. We performed multiple-group analyses with respect to gender and age after investigating the overall five-factor structure of the DBIQ-NL, because MG-CFA provides the opportunity to identify items that are non-invariant across groups. Invariance is a prerequisite for individual and group comparisons to reflect true differences [69, 74], not due to systematic differences in interpretation of items due to respondents’ group membership.

The extent of measurement invariance was evaluated in a series of three models. In model A, specifying ‘configural invariance’, the same factor structure is imposed on the two groups (formed by either sex or age)In the next model (B), specifying ‘weak invariance’, the factor loadings are constrained to be equal across groups. In Model C, ‘strong invariance’, the factor loadings *and* intercepts are constrained to be equal across groups. The model selection was performed by testing invariance by the Scaled Difference in Chi-Squares (SDCS) test [75] for nested models estimated with MLR. Inspection of size and consistency of factor loadings were performed to further evaluate model fit [69].

This sequential model estimation procedure to study measurement invariance is used since lack of strong factorial invariance will contaminate estimates of group mean differences [74]. It is widely acknowledged however, that the requirement of strong factorial invariance may be too strict and unrealistic a goal for group comparisons. Consequently, Byrne et al. [76] introduced the concept of partial invariance in which only a subset of parameters in each subscale must be invariant whereas others are allowed to vary between the groups. In this procedure, fit diagnostics (e.g., modification indices) can assist the researcher to identify specific items that are non-invariant across groups [69]. In our analysis partial invariance was investigated by inspecting modification indices to determine which cross-group equality constraint most significantly contributed to lack of fit; the model was re-estimated after freeing that constraint and this process was reiterated as needed [77]. Partial invariance of certain items signals qualitative group differences that render exact between-group comparisons with respect to subscales including these items possibly less meaningful. The importance of any violation of factorial invariance should be judged in relation to the intended use of the measure in practice [78] and may also be dependent on the number of affected items.

The implications of the findings with respect to measurement invariance are further investigated by comparing the original (sub-)scale scores to the adjusted (sub-)scale scores. Moreover, the correlations between the full and reduced scale and subscales were investigated, as well as the change in standardized factor loadings. Note that all (sub-)scale scores are calculated as average scores, which are unweighted and therefore not affected by (changes in) factor loadings.

## Results

Table 2 presents total mean scores and standard deviations in both DBIQ-NL samples as well as means and standard deviations for the questionnaires used for validation. Mean group differences between women and men and between younger and older participants are also included.

### Test-retest reliability

The intraclass correlation coefficients (ICC) between test and retest scores on the DBIQ-NL scale were .88 and on the DBIQ-NL subscales .82 for vitality, .80 for body acceptance, .78 for self-aggrandizement, .79 for sexual fulfillment, and .64 for physical contact. Test and retest scores were calculated using the original composition of the German version (Model 1).

### Construct validity

Pearson’s *r* between the DBIQ-NL and Body Cathexis Scale (BCS) correlated *r* = .60 (see Table 3). The subscale vitality of the DBIQ-NL correlated *r* = -.70 with the Checklist Individual Strength (CIS) and the subscale physical contact of the DBIQ-NL correlated *r* = .72 with the subscale comfort with physical touch of the Body Investment Scale (BIS). Correlation between DBIQ-NL and the Outcome Questionnaire (OQ-45) was -.51. DBIQ-NL and Rosenberg Self-esteem Scale (RSES) showed a correlation of *r* = .44. All correlations were in the expected direction and of medium to large size. Correlations between DBIQ-NL and BCS were notably higher for men than for women. Correlations between DBIQ-NL and OQ-45 and between DBIQ-NL and RSES were higher for age < 38 than for age ≥ 38. The subscale vitality correlated notably lower with the CIS in the younger group than in the older group. Correlations were calculated using the five-factor model identical to the original German version (Model 1). The correlations between the measures that were used for construct validation and the DBIQ-NL were recalculated (see Table 3) after the DBIQ-NL was revised on the basis of the CFA, as reported in the following section.

**Table 3:** Correlations in Pearson’s r of original and revised DBIQ scale and original and revised subscale ‘physical contact’ with validation instruments (Model 1 and Model 2).

	DBIQ-NL (35 items; model 1)					DBIQ-NL (31 items; model 2)				
	Total sample	female	male	young	old	Total sample	female	male	young	old
BCS^a^	.60	.53	.70	.58	.64	.59	.53	.67	.56	.64
OQ-45^b^	-.51	-.49	-.52	-.54	-.40	-.50	-.50	-.50	-.53	-.43
RSES^c^	.44	.38	.42	.49	.35	.45	.40	.43	.50	.37
	subscale physical contact (6 items)					subscale physical contact (4 items)				
BIS-touch^d^	.72	.77	.67	.71	.77	66	.71	.61	.64	.72
	subscale vitality					subscale vitality (as in Model 1)				
CIS^d^	-.70	-.67	-.73	-.66	-.83	-.70	-.67	-.73	-.66	-.83

Note: DBIQ-NL = Dresden Body Image Questionnaire, Dutch translation; OQ-45 = Outcome Questionnaire; RSES = Rosenberg Self-esteem Scale; BCS = Body Cathexis Scale; BIS-touch = subscale comfort with physical touch Body Investment Scale; CIS = Checklist Individual Strength.

For description of the samples 1a, 1b, and 2, see procedure and Table 2.

^a^ sample 1a

^b^ sample 2

^c^ samples 1b and 2

^d^ sample 1b

### Confirmatory factor analysis and measurement invariance on sample 1

First, a five-factor model identical to the original German version was specified (Model 1). Next, based on inspection of the modification indices of this model a five-factor model (Model 2) with item 33 (“I like showing my body”) loading on self-aggrandizement instead of body acceptance was evaluated. Goodness of fit indices of both models are shown in Table 4. The modification indices for correlated errors suggested partly the same (between reversed items 23 “I wish I had a different body” and 28 “If I could change something about my body, I would do it”), and partly different correlated errors (between reversed items 24 “I consciously avoid touching other people” and 19 “I do not like people touching me”), as reported by Pöhlmann et al. [26].

**Table 4:** DBIQ-NL: Confirmatory factor analysis, goodness of fit indices of Model 1, 2, and 3 and measurement invariance across sex and age of Model 2.

Model		*χ*^2^	*df*	*scl*	Δ *χ*^2^	Δ *df*	*T*_*s*_	*RMSEA (90% CI)*	*SRMR*	*CFI*	*TLI*
Model 1	Original 35 Item Scale	2169	550	1.265				.062 (.059-.065)	.070	.828	.814
Model 2	Adapted 35 Item Scale ^a^	1965	550	1.268				.058 (.055-.061)	.067	.850	.838
	Measurement Invariance Sex										
Model 2sA	configural invariance	2692	1100	1.268				.062 (.059-.065)	.074	.832	.818
Model 2sB	weak invariance	2721	1130	1.276	29	30	18.4	.061 (.058-.064)	.077	.832	.823
Model 2sC	strong invariance	2905	1160	1.322	184	30	60.2**	.063 (.060-.066)	.081	.816	.811
Model 2sC-1	partial strong item 19	2869	1158	1.317	148	28	49.8*	.062 (.060-.065)	.080	.819	.814
Model 2sC-2	partial strong items 19, 30	2827	1156	1.315	106	26	35.2*	.062 (.059-.065)	.079	.823	.818
Model 2sC-3	partial strong items 19, 30, 15	2804	1154	1.313	83	24	27.1	.061 (.059-.064)	.078	.826	.820
	Measurement Invariance Age										
Model 2aA	configural invariance	2623	1100	1.253				.060 (.057-.063)	.073	.842	.829
Model 2aB	weak invariance	2645	1130	1.247	22	30	21.4	.059 (.056-.062)	.075	.843	.835
Model 2aC	strong invariance	2796	1160	1.291	151	30	51.2**	.061 (.058-.064)	.078	.831	.826
Model 2aC-1	partial strong item 15	2771	1158	1.289	126	28	42.2*	.061 (.058-.063)	.078	.833	.828
Model 2aC-2	partial strong items 15, 28	2754	1156	1.286	109	26	36.5	.060 (.058-.064)	.077	.835	.830
Model 3	Reduced 31 Item Scale	1459	424	1.271	506	126	402.2**	.057 (.053-.060)	.063	.877	.865

Note: DBIQ-NL = Dresden Body Image Questionnaire, Dutch translation; *χ*^2^ = chi square; *df* = degrees of freedom; *scl* = scaling correction factor; *T*_*s*_ = Scaled.

Difference in Chi-Squares (SDCS) test statistic; *RMSEA* = root mean square error of approximation; *90% CI* = 90% confidence interval of the RMSEA; *SRMR* = standardized root mean square residual; *CFI* = comparative fit index; *TLI* = Tucker Lewis index.

^a^ Adapted model with item 33 not loading on the factor *acceptance* but on *self-aggrandizement* (modification index item 33 = 184.979).

*p < .01.

** p < .001.

The best fitting model (Model 2) was further evaluated for measurement invariance in a multiple group confirmatory factor analysis (MG-CFA) with sex and age as grouping variables. Models 2A-2C show measurement invariance tests for sex (2sA-2sC) and age (2aA-2aC) respectively. As can be seen with respect to sex as grouping variable, constraining the factor loadings to be equal across groups (2sB) is accompanied by only a slight decrease in CFI and a non-significant increase in chi square. Therefore, factor loadings can be considered as invariant across sex. In model 2sC factor loadings as well as intercepts are constrained to be equal across men and women. This restriction leads to a considerable loss of model fit, which may indicate that men and women interpret questionnaire items in different ways.

The same holds true for the model fits constraining factor loadings and intercepts to be equal across the two age groups: a model fit with only factor loadings constrained (2aB) leads to a non-significant increase in chi square. However, when both factor loadings and intercepts are constrained (2aC) a considerable loss of model fit is found.

Based on inspection of the modification indices, the following items were identified as contributing to lack of strong invariance across sex: item 19 (“I do not like people touching me”), item 30 (“I only allow a few people to touch me”), and item 15 (“I choose clothing that hides the shape of my body”). In model 2sC-3 the intercepts of these three items were freely estimated for men and women providing the best model fit for partial strong invariance across sex. On average, men score higher on these items than women (Table 2). With regard to age, items 15 (“I choose clothing that hides the shape of my body”) and 28 (“If I could change something about my body, I would do it”) were identified as contributing to lack of strong invariance. For item 15, the younger age group scores higher on average, whereas for item 28, the older age group scores higher. The partial strong invariance model represented in model 2aC-2 with the intercepts of these two items estimated freely represents the best fit.

### Fit of the revised model

A revised 31-item model, from which the 4 items (items 15, 19, 28, 30) primarily responsible for lack of invariance across sex and age were deleted, was evaluated. This model was fit to Sample 1 and reported as Model 3 in Table 4. Compared to the fit of the full 35-item model (Model 2), the revised 31-item model showed a better fit in all fit measures.

### Means of the revised subscales

Table 5 presents scores of the DBIQ-NL total scale and subscales for Model 2 as well as for the revised 31-item model, in which the four items contributing most to lack of invariance across sex and age were deleted.

**Table 5:** Scores on total scale DBIQ-NL and subscales based on Model 2, with and without items 15, 28 (subscale body acceptance), 19, and 30 (subscale physical contact).

		Full sample		Women		Men			age < 38		age ≥ 38		
		*n =* 761		*n* = 433		*n* = 326			*n* = 540		*n* = 221		
*n* items	(sub)scale (items)	Mean	*SD*	Mean	*SD*	Mean	*SD*	*d*	Mean	*SD*	Mean	*SD*	*d*
35	DBIQ-NL	3.66	0.45	3.56	0.44	3.79	0.43	0.53	3.71	0.44	3.53	0.45	0.40
31	DBIQ-NL	3.65	0.45	3.56	0.44	3.77	0.44	0.48	3.71	0.44	3.51	0.46	0.44
8	Vitality (2,3,6,8,14,17,26,32)	3.87	0.58	3.76	0.58	4.02	0.55	0.46	3.91	0.55	3.78	0.66	0.21
7	Body Acc. (7,12,15,18,23,25,28)	3.83	0.67	3.70	0.69	4.00	0.60	0.46	3.85	0.67	3.79	0.68	0.09
5	Body Acc. (7,12,18,23,25)*	3.87	0.69	3.74	0.71	4.03	0.62	0.44	3.89	0.68	3.82	0.69	0.10
8	Self-aggr. (1,10,13,20,29,31,33,34)	3.12	0.55	3.06	0.56	3.19	0.56	0.23	3.23	0.53	2.86	0.52	0.70
6	Phys. Contact (5, 11, 19, 22, 24, 30)	3.80	0.60	3.76	0.61	3.86	0.59	0.17	3.84	0.59	3.71	0.62	0.21
4	Phys. Contact (5, 11, 22, 24)*	3.85	0.61	3.87	0.60	3.81	0.60	-0.10	3.89	0.59	3.75	0.63	0.23
6	Sexual Fulfillment (4,9,16,21,27,35)	3.78	0.71	3.60	0.72	4.01	0.64	0.60	3.83	0.69	3.65	0.74	0.25

Note: DBIQ-NL = Dresden Body Image Questionnaire, Dutch translation; Self-aggr. = Self-aggrandizement; Body Acc. = Body Acceptance; Phys. Contact = Physical Contact

* Adapted scale with items removed based upon MGCFA outcomes.

Total mean scores were hardly influenced by deleting items 15, 19, 28, and 30 with changes of less than 0.02 in size. The correlation between the full and reduced scale was .99. Scores on the subscale body acceptance (with items 15 and 28 deleted) changed at most 0.04 points. The correlation between the full and reduced sub-scale was .96. Mean scores for the subscale physical contact increased slightly (.05) for the whole group when items 19 and 30 were deleted; the correlation between the original and adapted sub-scale was .92; Mean scores were .11 higher for women and showed a decrease of .05 for men. Mean differences on the subscales between age groups and between women and men were small to medium (Cohen’s d <. 0.50), with the exception of medium differences between women and men on the subscale sexual fulfillment (*d* = 0.60) and between the younger and older age group on the subscale self-aggrandizement (*d* = 0.70). Differences were in the same direction, with men scoring higher than women and the younger age group scoring higher than the older age group. The only exception were men scoring slightly lower than women on the reduced physical contact subscale (*d* = 0.10). For standardized factor loadings of the full and reduced item sub-scales see S3 Table.

## Discussion

### Structure of the (sub)scales

The aim of this study was to assess psychometric properties of the DBIQ in a non-clinical Dutch sample. Moreover, the work done by the developers of the original scale [26, 27] was extended by evaluating the equivalence of the measurement model across sex and age. For our evaluation of the psychometric properties we used a large convenience sample with a gender and age distribution deviating from the Dutch general population. Whereas the investigation of age invariance of the DBIQ was in fact facilitated by the bimodal age distribution, the sample does not provide information on the distribution of DBIQ scores across the general population (e.g. with the purpose of computing norm scores).

Confirmatory Factor Analysis based on the five-factor structure of the original German questionnaire, showed adequate fit in the translated version for RMSEA and SRMR, but mediocre fit for CFI and TLI [69]. Adaptation of the model based on inspection of the modification indices led to improvement of model fit. In this adaptation, the item “I like showing my body” was moved from the subscale body acceptance to the subscale self-aggrandizement. This is in line with the results of the principal component analysis by Probst et al. [34], which revealed higher loadings on the subscale self-aggrandizement for the item “I like showing my body” than on the original subscale. Even though German and Dutch are closely related languages, a slight semantic difference may have occurred, suggesting that the Dutch translation emphasizes the pleasure of showing one’s body, whereas the original item was meant to measure body acceptance. A semantic explanation is more plausible than a cultural difference in the interpretation of this item.

Based on the fit indices and considering that reallocating one item is an acceptable model change when an instrument is used in a different cultural setting or language [61], the adapted model was used for further evaluations. After comparing the quality of the goodness of fit indices, as recommended by [79], with findings from previous research based on the German inventory, we judge the psychometric properties of the DBIQ-NL as sufficient to warrant further research on the scale and its properties, in a Dutch clinical population. While being aware of new developments in scale analysis by Exploratory Structural Equation Modeling to remedy the overly strict CFA requirements [68, 80], we refrained from applying this method in order to obtain a close comparison between the DBIQ-NL and the original DBIQ by following a similar evaluation procedure. Based on [67, 79], it was to be expected that the DBIQ-NL would show a reduced goodness of fit compared to the original scale, in which the reported CFI only just met the often used threshold of 0.90.

A multiple-group confirmatory factor analysis showed configural invariance. The equal item intercepts hypothesis, implying factorial invariance, however, was not supported for all subscales across sex and age. When testing partial factorial invariance, four items were identified that contributed to the lack of strong invariance across sex (item 19, 30) or age (item 28) or across both groups (item 15) indicating that interpretation of the meaning of these items and therefore the item scores might depend on group membership. Replication of the procedure by fitting a final model in sample 1 without these items supported the best, although moderate, fit of this 31-item scale.

Next to the technical identification of these differing items, there are arguments from a qualitative perspective as to why these items differ across groups. Some items suggest interesting patterns. In items 19 and 30, differing across sex, being touched by others is the central issue. These items may be interpreted differently by men and women since the latter are more often the object of undesired physical contact. Invariance across sex in item 15 (“I choose clothing that hides the shape of my body”) could be explained by the fact that for men, in contrast to women, clothing is not a well-known strategy to camouflage less accepted parts of the body [81]. The differences across age for this item might be indicative for age- or cohort-related cultural differences with regard to showing the body. Item 28 (“If I could change something about my body, I would do it”) might mean something different for the older age group, in light of physical impairments or other confrontations with unwelcome bodily changes. Millsap and Kwok [78] describe the option of using a shortened version of the scale that omits the items that performed differently across groups, at the same time warning that this option results in as many versions of the scale as there are invariance studies.

A second option is to retain all items when the net impact of the differences on the total score is small [78]. This is indeed the case in the present non-clinical sample. Also due to the limited number of affected items, partial invariance only slightly affects the differences between sex and age groups in mean total scores on the DBIQ-NL and subscales. Therefore, adaptations need not be made when the scale is used to gain global information on body image in non-clinical groups. However, when the focus is on gathering information with respect to differences on the issue of physical contact across the sexes or on the theme of body acceptance among different age groups, a score without the items violating invariance could be relevant.

### Reliability and validity

The DBIQ-NL and its five subscales showed adequate internal consistency. Test-retest reliability was also adequate, although based on a rather small sample. Good construct validity was found with moderate associations with body satisfaction (BCS) as expected since the DBIQ aims to measure a broad concept of body image whereas in the BCS appearance-related issues are prominent. The higher correlations between total score on the DBIQ and body satisfaction (BCS) for men than for women can possibly be explained by this difference since earlier studies found that women are likely to give a greater emphasis than men to appearance as part of the concept body image [30, 82]. The moderate associations between the DBIQ-NL and the OQ-45 and RSES confirm the relation between body image, psychosocial well-being and self-esteem and its importance when assessing non-clinical samples [5, 6].

Correlations between the subscales were relatively small, providing support for the multidimensionality of the instrument. Validation of the subscales for physical contact and vitality by correlating these with other indices showed good construct validity.

### Implications for the use of the DBIQ-NL and further research

The present study on the psychometric properties of the DBIQ in a Dutch non-clinical sample shows a factor structure and model fit that are satisfactory and motivate further study. In view of our aim to make an instrument available in Dutch language measuring body image as a multifaceted construct in a non-clinical as well as in a clinical population, future research will have to address a variety of clinical samples. These further evaluations should also pay attention to measurement invariance across sex and age.

Another issue of importance in clinical samples is the intimate and for a substantial group of patients possibly trauma related nature of the subscale sexual fulfillment. Applicability of this subscale still needs to be evaluated. On the other hand, because both clinicians and patients seem to be reluctant to discuss issues concerning sexuality, a reluctance that may also be related to cultural or religious background, addressing sexuality in a self-report questionnaire may also make it easier for both client and therapist to pay attention to this relevant theme [28].

A recent study using the DBIQ-NL in a broad clinical sample including mood disorders, anxiety disorders, adjustment disorder, post-traumatic stress disorder, eating disorders, schizophrenia and other psychotic disorders [83] gave a first indication of the clinical usefulness of the scale. The study suggests that body image is a common problem occurring in most patients with mental disorders. Body acceptance and sexual fulfillment were the most differentiating aspects of body image between diagnoses. Surprisingly, vitality did not differ significantly between the various disorders. It may be concluded that the DBIQ has potential surplus value in the field of body image since it covers a broad range of body image dimensions and includes important dimensions such as touch and sexuality that are not well represented in other measures of body image.

## Supporting information

S1 Fig Distribution of age in the two samples. (DOCX)

S1 Table Dresden Body Image Questionnaire (DBIQ), Dutch version. (DOCX)

S2 Table DBIQ-NL: Item means and standard deviations sample 1; item means and standard deviations per sex and age group sample 1. (DOCX)

S3 Table Standardized factor loadings full and reduced item sub-scales. (DOCX)

S1 Waiver Ethical Approval (PDF)

S1 Datafile Sample 1. (SAV)

S2 Datafile Sample 2. (SAV)
