# Supplementary figures and images for: Psychometric properties of the Dresden Body Image Questionnaire: A multiple-group confirmatory factor analysis across sex and age in a Dutch non-clinical sample
Source: PLoS One. 2017 Jul 26;12(7):e0181908. doi: 10.1371/journal.pone.0181908 (PMC5528876; doi:10.1371/journal.pone.0181908)

Supplement 1 Figure. Distribution of Age in the Two Samples.
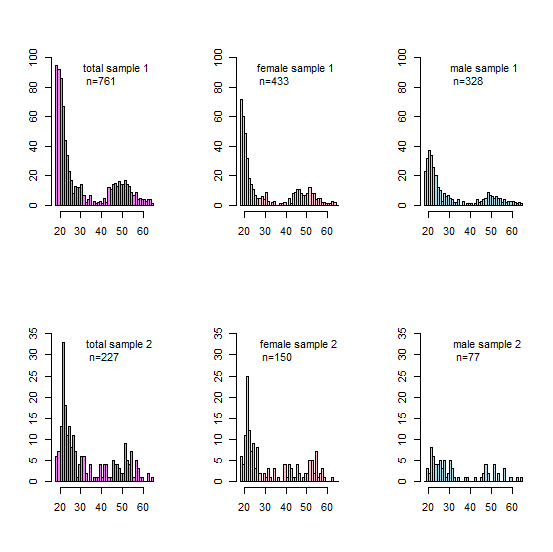

Supplement: S1 Fig — (DOCX) [file pone.0181908.s001.docx]
