# Supplementary material for: Psychometric properties of the Dresden Body Image Questionnaire: A multiple-group confirmatory factor analysis across sex and age in a Dutch non-clinical sample
Source: PLoS One. 2017 Jul 26;12(7):e0181908. doi: 10.1371/journal.pone.0181908 (PMC5528876; doi:10.1371/journal.pone.0181908)
Supplement: S1 Table — (DOCX) [file pone.0181908.s002.docx]

**S1 Table. Dresden Body Image Questionnaire (DBIQ), Dutch version.**

| 1.a | Ik beweeg me sierlijk. |
| --- | --- |
| 2.v | Ik voel me vaak lichamelijk niet gezond. (R) |
| 3.v | Ik heb geen veerkracht. (R) |
| 4.s | Tijdens seksualiteit beleef ik mijn lichaam intens en als aangenaam. |
| 5.p | Lichamelijk contact vind ik belangrijk om nabijheid te laten blijken. |
| 6.v | Ik voel me lichamelijk vaak slap. (R) |
| 7.a | In veel situaties ben ik tevreden met mijn lichaam. |
| 8.v | Ik ben lichamelijk fit. |
| 9.s | Ik ben zeer tevreden met mijn seksuele beleving. |
| 10.a | Andere mensen vinden mij aantrekkelijk. |
| 11.p | Ik zoek lichamelijke nabijheid en tederheid op. |
| 12.a | Ik hou van mijn lichaam. |
| 13.a | Ik vind het prettig en spannend wanneer iemand met aandacht naar me kijkt. |
| 14.v | Ik heb veel energie. |
| 15.a | Ik kies mijn kleding bewust zo uit dat deze mijn lichaam verbergt. (R) |
| 16.s | Seksualiteit is voor mij een belangrijk deel van mijn leven. |
| 17.v | Ik ben lichamelijk tot veel in staat. |
| 18.a | Ik voel me vaak onprettig in mijn lichaam. (R) |
| 19.p | Ik hou er niet van wanneer iemand mij aanraakt. (R) |
| 20.a | Wanneer iemand aandacht aan mijn lichaam schenkt, voel ik me gewaardeerd. |
| 21.s | Ik kan zonder remming van seksuele situaties genieten. |
| 22.p | Ik vind het prettig wanneer iemand me omarmt. |
| 23.a | Ik zou graag een ander lichaam hebben. (R) |
| 24.p | Ik vermijd het bewust om andere mensen aan te raken. (R) |
| 25.a | Ik ben tevreden met mijn uiterlijk. |
| 26.v | Ik bereik lichamelijk snel mijn grenzen. (R) |
| 27.s | Ik kan van mijn seksualiteit genieten. |
| 28.a | Als ik iets aan mijn lichaam zou kunnen veranderen, zou ik het doen. (R) |
| 29.a | Mijn lichaam is expressief. |
| 30.p | Lichamelijke aanraking laat ik maar van weinig mensen toe. (R) |
| 31.a | Ik zet mijn lichaam in om aandacht te krijgen. |
| 32.v | Ik ben lichamelijk belastbaar en kan tegen een stootje. |
| 33.a | Ik toon mijn lichaam graag. |
| 34.a | Ik sta graag in het middelpunt van de belangstelling. |
| 35.s | Mijn seksuele ervaringen zijn bevredigend voor mij. |

Note: R = scored in the reversed direction. a = subscale self-aggrandizement; b = subscale body acceptance; p = subscale physical contact; s = subscale sexual fulfillment; v = subscale vitality.
