# Supplementary material for: Psychometric properties of the Dresden Body Image Questionnaire: A multiple-group confirmatory factor analysis across sex and age in a Dutch non-clinical sample
Source: PLoS One. 2017 Jul 26;12(7):e0181908. doi: 10.1371/journal.pone.0181908 (PMC5528876; doi:10.1371/journal.pone.0181908)
Supplement: S2 Table — (DOCX) [file pone.0181908.s003.docx]

**S2 Table. DBIQ-NL: Item Means and Standard Deviations Sample 1; Item Means and**

**Standard Deviations per Sex and Age Group Sample 1.**

|  | Sample 1 | | |  | Sample 1 Women | | |  | Sample 1 Men | | |  | Sample 1 age < 38 | | |  | Sample 1 age ≥ 38 | | |
| --- | --- | --- | --- | --- | --- | --- | --- | --- | --- | --- | --- | --- | --- | --- | --- | --- | --- | --- | --- |
|  | n | Mean | SD |  | n | Mean | SD |  | n | Mean | SD |  | n | Mean | SD |  | n | Mean | SD |
| Item 1 | 759 | 3.26 | 0.90 |  | 432 | 3.26 | 0.89 |  | 325 | 3.26 | 0.91 |  | 539 | 3.28 | 0.90 |  | 220 | 3.20 | 0.88 |
| Item 2 (R) | 760 | 3.95 | 0.84 |  | 432 | 3.87 | 0.86 |  | 326 | 4.06 | 0.79 |  | 540 | 3.96 | 0.81 |  | 220 | 3.93 | 0.89 |
| Item 3 (R) | 757 | 3.90 | 0.99 |  | 432 | 3.82 | 0.90 |  | 323 | 4.00 | 0.93 |  | 537 | 3.93 | 0.86 |  | 220 | 3.81 | 1.04 |
| Item 4 | 755 | 3.91 | 0.92 |  | 429 | 3.71 | 0.89 |  | 324 | 4.17 | 0.84 |  | 535 | 3.93 | 0.86 |  | 220 | 3.87 | 0.98 |
| Item 5 | 756 | 3.84 | 0.83 |  | 430 | 3.85 | 0.81 |  | 324 | 3.82 | 0.86 |  | 537 | 3.83 | 0.84 |  | 219 | 3.87 | 0.81 |
| Item 6 (R) | 759 | 3.89 | 0.81 |  | 432 | 3.81 | 0.81 |  | 325 | 3.99 | 0.79 |  | 539 | 3.86 | 0.79 |  | 220 | 3.95 | 0.85 |
| Item 7 | 759 | 3.82 | 0.80 |  | 433 | 3.73 | 0.83 |  | 324 | 3.94 | 0.74 |  | 540 | 3.83 | 0.79 |  | 219 | 3.81 | 0.80 |
| Item 8 | 761 | 3.89 | 0.83 |  | 433 | 3.78 | 0.84 |  | 326 | 4.04 | 0.80 |  | 540 | 3.94 | 0.82 |  | 221 | 3.78 | 0.85 |
| Item 9 | 754 | 3.80 | 0.94 |  | 427 | 3.66 | 1.00 |  | 325 | 3.98 | 0.88 |  | 535 | 3.88 | 0.90 |  | 219 | 3.61 | 1.00 |
| Item 10 | 758 | 3.43 | 0.69 |  | 431 | 3.41 | 0.67 |  | 325 | 3.46 | 0.73 |  | 538 | 3.49 | 0.70 |  | 220 | 3.30 | 0.65 |
| Item 11 | 759 | 3.47 | 0.85 |  | 433 | 3.50 | 0.85 |  | 324 | 3.44 | 0.85 |  | 538 | 3.52 | 0.83 |  | 221 | 3.36 | 0.87 |
| Item 12 | 758 | 3.56 | 0.85 |  | 431 | 3.43 | 0.89 |  | 325 | 3.72 | 0.79 |  | 537 | 3.57 | 0.86 |  | 221 | 3.53 | 0.83 |
| Item 13 | 759 | 3.62 | 0.89 |  | 433 | 3.50 | 0.91 |  | 324 | 3.77 | 0.85 |  | 538 | 3.73 | 0.86 |  | 221 | 3.33 | 0.91 |
| Item 14 | 759 | 3.79 | 0.81 |  | 432 | 3.69 | 0.84 |  | 325 | 3.93 | 0.75 |  | 538 | 3.84 | 0.77 |  | 221 | 3.68 | 0.89 |
| Item 15 (R) | 757 | 3.88 | 0.98 |  | 431 | 3.65 | 0.99 |  | 324 | 4.18 | 0.88 |  | 538 | 4.00 | 0.93 |  | 219 | 3.59 | 1.03 |
| Item 16 | 759 | 3.46 | 0.99 |  | 433 | 3.28 | 0.95 |  | 324 | 3.70 | 0.94 |  | 538 | 3.56 | 0.95 |  | 221 | 3.22 | 0.96 |
| Item 17 | 761 | 3.90 | 0.79 |  | 433 | 3.73 | 0.81 |  | 326 | 4.12 | 0.67 |  | 540 | 3.97 | 0.75 |  | 221 | 3.73 | 0.86 |
| Item 18 (R) | 757 | 4.06 | 0.90 |  | 429 | 3.88 | 0.90 |  | 326 | 4.29 | 0.83 |  | 538 | 4.06 | 0.87 |  | 219 | 4.06 | 0.96 |
| Item 19 (R) | 759 | 4.05 | 0.91 |  | 433 | 3.89 | 0.89 |  | 324 | 4.25 | 0.89 |  | 539 | 4.12 | 0.85 |  | 220 | 3.88 | 1.03 |
| Item 20 | 760 | 3.68 | 0.84 |  | 433 | 3.62 | 0.84 |  | 325 | 3.74 | 0.85 |  | 540 | 3.80 | 0.76 |  | 220 | 3.36 | 0.85 |
| Item 21 | 756 | 3.67 | 1.00 |  | 430 | 3.45 | 1.00 |  | 324 | 3.98 | 0.91 |  | 537 | 3.70 | 1.00 |  | 219 | 3.59 | 0.99 |
| Item 22 | 754 | 3.93 | 0.83 |  | 428 | 3.98 | 0.82 |  | 324 | 3.86 | 0.84 |  | 537 | 3.99 | 0.84 |  | 217 | 3.79 | 0.80 |
| Item 23 (R) | 755 | 4.15 | 1.00 |  | 429 | 3.99 | 1.04 |  | 324 | 4.34 | 0.92 |  | 538 | 4.17 | 0.98 |  | 217 | 4.10 | 1.05 |
| Item 24 (R) | 759 | 4.15 | 0.87 |  | 432 | 4.17 | 0.86 |  | 325 | 4.12 | 0.88 |  | 540 | 4.21 | 0.80 |  | 219 | 3.99 | 1.00 |
| Item 25 | 761 | 3.76 | 0.77 |  | 433 | 3.68 | 0.79 |  | 326 | 3.87 | 0.73 |  | 540 | 3.82 | 0.73 |  | 221 | 3.62 | 0.85 |
| Item 26 (R) | 758 | 3.66 | 0.92 |  | 431 | 3.56 | 0.90 |  | 325 | 3.79 | 0.93 |  | 538 | 3.73 | 0.89 |  | 220 | 3.49 | 0.97 |
| Item 27 | 749 | 3.92 | 0.89 |  | 428 | 3.75 | 0.93 |  | 319 | 4.16 | 0.77 |  | 533 | 3.98 | 0.87 |  | 216 | 3.78 | 0.93 |
| Item 28 (R) | 760 | 3.59 | 1.19 |  | 433 | 3.53 | 1.19 |  | 325 | 3.67 | 1.20 |  | 540 | 3.50 | 1.19 |  | 220 | 3.81 | 1.18 |
| Item 29 | 757 | 3.26 | 0.82 |  | 431 | 3.24 | 0.81 |  | 324 | 3.29 | 0.83 |  | 537 | 3.35 | 0.79 |  | 220 | 3.06 | 0.86 |
| Item 30 (R) | 758 | 3.33 | 1.06 |  | 432 | 3.16 | 1.05 |  | 324 | 3.56 | 1.03 |  | 538 | 3.36 | 1.07 |  | 220 | 3.28 | 1.04 |
| Item 31 | 758 | 2.25 | 0.93 |  | 430 | 2.20 | 0.92 |  | 326 | 2.31 | 0.94 |  | 537 | 2.40 | 0.92 |  | 221 | 1.89 | 0.86 |
| Item 32 | 761 | 3.94 | 0.83 |  | 433 | 3.77 | 0.85 |  | 326 | 4.15 | 0.75 |  | 540 | 4.00 | 0.79 |  | 221 | 3.79 | 0.91 |
| Item 33 | 760 | 2.83 | 0.91 |  | 433 | 2.76 | 0.92 |  | 325 | 2.92 | 0.89 |  | 539 | 2.97 | 0.88 |  | 221 | 2.48 | 0.88 |
| Item 34 | 757 | 2.65 | 1.00 |  | 432 | 2.52 | 0.96 |  | 323 | 2.81 | 1.03 |  | 537 | 2.79 | 1.00 |  | 220 | 2.30 | 0.92 |
| Item 35 | 755 | 3.77 | 0.94 |  | 429 | 3.66 | 0.97 |  | 324 | 3.91 | 0.89 |  | 535 | 3.79 | 0.94 |  | 220 | 3.71 | 0.96 |

Note: DBIQ-NL = Dresden Body Image Questionnaire, Dutch translation.
