# Supplementary material for: Psychometric properties of the Dresden Body Image Questionnaire: A multiple-group confirmatory factor analysis across sex and age in a Dutch non-clinical sample
Source: PLoS One. 2017 Jul 26;12(7):e0181908. doi: 10.1371/journal.pone.0181908 (PMC5528876; doi:10.1371/journal.pone.0181908)
Supplement: S3 Table — (DOCX) [file pone.0181908.s004.docx]

| S3 Table. Standardized factor loadings full and reduced item sub-scales. | | |
| --- | --- | --- |
|  | Original scale | Revised  scale |
| Sub-scale  Body acceptance |  |  |
| Item 23 | 0.745 | 0.715 |
| Item 15 | 0.525 | - |
| Item 18 | 0.726 | 0.709 |
| Item 12 | 0.769 | 0.783 |
| Item 7 | 0.733 | 0.738 |
| Item 28 | 0.502 | - |
| Item 25 | 0.712 | 0.718 |
| Sub-scale  Physical contact |  |  |
| Item 11 | 0.734 | 0.830 |
| Item 24 | 0.509 | 0.371 |
| Item 22 | 0.542 | 0.514 |
| Item 19 | 0.546 | - |
| Item 30 | 0.515 | - |
| Item 5 | 0.596 | 0.633 |
