# Supplementary material for: Psychometric properties of the Dresden Body Image Questionnaire: A multiple-group confirmatory factor analysis across sex and age in a Dutch non-clinical sample
Source: PLoS One. 2017 Jul 26;12(7):e0181908. doi: 10.1371/journal.pone.0181908 (PMC5528876; doi:10.1371/journal.pone.0181908)
Supplement: S1 Waiver Ethical Approval — (PDF) [file pone.0181908.s005.pdf]

De Boelelaan 1117
1081 HV Amsterdam

postbus 7057
1007 MB Amsterdam

telefoon 020 444 4444

www.VUmc.nl

Medisch Ethische Toetsingscommissie
VU medisch centrum
voorzitter: prof. dr. J.A. Rauwerda
intern postadres: BS7, kamer H-565
telefoon: 020 - 44 45585
e-mail: metc@vumc.nl
website: www.vumc.nl/metc

dr. R.J. Bosscher
voormalig Universitair docent
VU/ Hogeschool Windesheim, kamer A220
8000 GB ZWOLLE

VUmc

onderwerp	ons kenmerk	datum
niet-WMO advies	2016.426	14 september 2016

Geachte heer Bosscher,

Het Dagelijks Bestuur van de Medisch Ethische Toetsingscommissie VU medisch centrum heeft uw onderzoek **Psychometrische evaluatie van de Dresden Body Image Questionnaire** besproken in de vergadering van 13/09/2016.

Het onderzoek valt niet onder de reikwijdte van de Wet Medisch-wetenschappelijk Onderzoek met mensen (WMO). Dit oordeel geldt met terugwerkende kracht tot 01/01/2011.

Het oordeel is gebaseerd op de volgende documenten:

Sectie	Onderwerp	Versie
A1	aanbiedingsbrief	bijlage: vraag tijdschrift
A1	aanbiedingsbrief	d.d. 6-9-2016
B25	privacyverklaring	d.d. 6-9-2016
C1	onderzoeksprotocol	versie 1 d.d. 6-9-2016
F1	vragenlijst	Body Cathexis Scale versie 2 met loge d.d. 6-9-2016
F1	vragenlijst	Body Investment Scale, subschaal aanraking versie 1
F1	vragenlijst	Checklist Individuele Spankracht versie 1 d.d. 6-9-2016
F1	vragenlijst	Rosenberg Self Esteem schaal versie 1 d.d. 6-9-2016
F1	vragenlijst	versie 1 DKB-35 Lichaamsbeleving

Het Dagelijks Bestuur van de Medisch Ethische Toetsingscommissie VU medisch centrum wijst u erop dat hoewel het ingediende onderzoek niet onder de reikwijdte van de WMO valt, andere wet- en regelgeving (mogelijk) wel van toepassing is, waaronder:

- WGBO (Wet Geneeskundige BehandelingsOvereenkomst);
- WBP (Wet Bescherming Persoonsgegevens), zie www.cbpweb.nl;
- Code Goed Gedrag (Gedragscode gezondheidsonderzoek: gebruik medische gegevens in wetenschappelijk onderzoek), zie www.federa.org;
- Code Goed Gebruik (Gedragscode Verantwoord omgaan met lichaamsmateriaal ten behoeve van wetenschappelijk onderzoek, 2011), zie www.federa.org;
- Reglement VUmc Nader gebruik lichaamsmateriaal, zie KwaliteitsNet VUmc, document 046913, versie 2;

202.000.157/2

- Overdrachtsovereenkomst van lichaamsmateriaal, zie http://www.vumc.nl/afdelingen/METc/wetgeving/lichaamsmateriaal/;
- Biobanken: Tijdelijke regeling 'de novo' biobanken (METc VUmc 2012), zie http://www.vumc.nl/afdelingen/METc/wmo-oordeel/soorten_onderzoek/tijdelijke_regeling_biobanken/;
- WBO (Wet Bevolkings Onderzoek), zie http://www.vumc.nl/afdelingen/METc/wetgeving/wetbevolkingsonderzoek/.

To whom it may concern

We are pleased to confirm that the Medical Research Involving Human Subjects Act (WMO) does not apply to the above mentioned study and that an official approval of this study by our committee is not required. This approval is retroactive as of 01/01/2011.

The Medical Ethics Review Committee of VU University Medical Center is registered with the US Office for Human Research Protections (OHRP) as IRB00002991. The FWA number assigned to VU University Medical Center is FWA00017598.

Met vriendelijke groet,
namens de Medisch Ethische Toetsingscommissie VU medisch centrum,

p.o.

prof. dr. J.A. Rauwerda, voorzitter

c.c.: drs. W.J. Scheffers / wj.scheffers@windesheim.nl
